# Five alternative *Helicobacter pylori* antibiotics to counter high levofloxacin and metronidazole resistance in the Dominican Republic

**DOI:** 10.1371/journal.pone.0213868

**Published:** 2019-03-27

**Authors:** Muhammad Miftahussurur, Modesto Cruz, Dalla Doohan, Phawinee Subsomwong, José A. Jiménez Abreu, Celso Hosking, Langgeng Agung Waskito, Yoshio Yamaoka

**Affiliations:** 1 Gastroentero-Hepatology Division, Department of Internal Medicine, Faculty of Medicine-Institute of Tropical Disease, Universitas Airlangga, Surabaya, Indonesia; 2 Institute of Tropical Disease, Universitas Airlangga, Surabaya, Indonesia; 3 Institute of Microbiology and Parasitology, Faculty of Science, Autonomous University of Santo Domingo, Santo Domingo, Dominican Republic; 4 Department of Biomedical Research, National Institute of Medicine and Diagnostic Imaging, Santo Domingo, Dominican Republic; 5 Department of Environmental and Preventive Medicine, Oita University Faculty of Medicine, Yufu, Japan; 6 Dominican–Japanese Digestive Disease Center, Dr. Luis E. Aybar Health and Hygiene City, Santo Domingo, Dominican Republic; 7 Department of Medicine, Gastroenterology and Hepatology Section, Baylor College of Medicine, Houston, Texas, United States of America; 8 Faculty of Medicine, Universitas Airlangga, Surabaya, Indonesia; Nitte University, INDIA

## Abstract

The prevalence of *Helicobacter pylori* resistance to levofloxacin and metronidazole was high in the Dominican Republic. We used two-fold agar dilution method to determine the minimum inhibitory concentration of five alternative antibiotics in 63 Dominican strains. We also assessed the genetic mutations associated with the antibiotic resistance using next-generation sequencing. We revealed that all 63 strains were sensitive towards sitafloxacin, furazolidone, and rifabutin. In contrast, the prevalence of rifaximin and garenoxacin resistance were high (82.5% and 34.9%, respectively). Patients more than or equal to 60 years old had the highest risk of double-antibiotic resistance (7/9, 77.8%, OR = 31.5, P = 0.009) and garenoxacin resistances (8/9, 88.9%, OR = 45.33, P = 0.002) with an increasing risk simultaneously by age (P = 0.004, r = 0.357). Almost all rifaximin resistant strains possessed multiple mutations with more than three mutations within *rpoB* including the most frequent novel mutations of S352L, I2726L, and V2465A. There was a significant association between *vacA* genotype and rifaximin resistance (P = 0.042). Among 23 levofloxacin-resistant strains, 82.6% (19/23, P <0.001) were also resistant to garenoxacin, and 39.1% (9/23) had a high minimal inhibitory concentration ≥8 μg/mL with positive trend correlation (P = <0.001, r = 0.84). Among 19 garenoxacin resistant strains, 16 (84.2%) contained mutations at D91 and N87 of *gyrA*. In conclusion, sitafloxacin, rifabutin, and furazolidone might be considered as alternative antibiotics to be included in *H*. *pylori* eradication regimen in regions with high prevalence of levofloxacin and metronidazole resistance, such as the Dominican Republic.

## Introduction

*Helicobacter pylori*, causative agent in the pathogenesis of gastroduodenal disease including gastric cancer, highly colonized Latin American populations thus brought this region to be the second highest infection rate worldwide after African populations. [1] In addition, Latin America is a region with significant burden of gastric cancer (9.7 cases per 100,000 people per year; GLOBOCAN 2012; http://globocan.iarc.fr). Moreover, meta-analysis reported a higher recurrence rate of *H*. *pylori* infection in this region (7.9, 11.2, and 6.2 cases per 100 person-years, first year and in subsequent years, respectively) than the estimated global (4.5/100 person-years) [2]. Several countries in Latin America showed an emerging *H*. *pylori* antibiotics resistance rate; including 16% for clarithromycin, 33% for tetracycline and 83% for metronidazole in Colombia, 15% for amoxicillin in Brazil, and 37% for levofloxacin in Peru [3].

The Dominican Republic is a sovereign state located in the Caribbean Sea region with an approximately total population of 11 million, located in the western portion of Hispaniola Island. The population density is the highest in the southern area, where the capital city of Santo Domingo is located (https://www.cia.gov/). Although the gastric cancer risk in the Dominican Republic is reported to be low (7.3 per 100,000 year^–1^ based on http://globocan.iarc.fr/), the infection rate of *H*. *pylori* was reported to be high (58.9%) [4]. In addition, *H*. *pylori* contained virulent types (*cagA*-positive/*vacA* s1m1) were predominant, which induced more severe gastritis [4]. Previously, we reported that clarithromycin and amoxicillin resistance against *H*. *pylori* in the Dominican Republic strains were low (3.1% and 1.6%, respectively) [5]. Maastricht V consensus for management of *H*. *pylori* infection mentioned that clarithromycin-based therapy should be abandoned in case the clarithromycin resistance rate in the region is more than 15% [6]. Thus, we recommended that initial treatment for *H*. *pylori* infection with clarithromycin-based standard triple therapy might still applicable in the Dominican Republic [5]. Nevertheless, we have to take an important note that the emerging resistance to metronidazole and levofloxacin were demonstrated (82.8% and 35.9%, respectively). Both antibiotics-based regimens would be insufficient as primary or secondary *H*. *pylori* antibiotic regimens in the Dominican Republic [5]. Other appropriate antibiotics including novel regimens are necessary to counter multiple side effects due to a repeated treatment course.

Furazolidone is a synthetic nitrofuran derivative which is utilized for an alternative drug with high efficacy but low cost for *H*. *pylori* eradication therapy as a component of the multidrug regimens, particularly in association with bismuth [7, 8]. Novel quinolones become potential candidate agents including garenoxacin and sitafloxacin, which are known to have high affinity to DNA gyrase and low minimum inhibitory concentration (MIC) against *H*. *pylori*, thus superior rather than levofloxacin-based regimen, especially against *gyrA* mutations [9]. Rifaximin, a semisynthetic rifamycin derivate which act as a surface antibiotic against *H*. *pylori* including clarithromycin-resistant strain. It becomes alternative drug due to rare primary resistance, poor absorbance to the blood and the bactericidal effect is not significantly affected by an acidic environment [10]. Rifabutin, the rifamycin-S derivate which formed spiro-piperidyl-rifamycin [11], become a promising *H*. *pylori* treatment which demonstrated high in vitro activity, chemically stable and not share resistance to clarithromycin [12]. Mutation in the *rpoB* gene was reported to have association with the *H*. *pylori* resistance of rifabutin and rifaximin, which mostly encoded the DNA-directed RNA polymerase [13].

The Dominican Republic could be used as a model of the country with high metronidazole and levofloxacin resistances. Additionally, while our previous study mentioned the low amoxicillin and clarithromycin resistance rates, there was no study investigating the susceptibility of alternative antibiotic to combat *H*. *pylori* infection in Dominican Republic [5]. Providing an update of alternative antibiotic susceptibility data might give more options for clinician to determine the therapy. Therefore, further investigation about an alternative antibiotic therapy is important to decide better regimen especially after the first-line, amoxicillin or clarithromycin-based therapy failure. The aim of the present study was to examine the susceptibility of five alternative antibiotics to the *H*. *pylori* isolates from the Dominican Republic. We also analyzed the DNA sequences of genes involved in antibiotic resistance to identify candidate mutations that might play a role in the antibiotic resistance mechanism.

## Materials and methods

### Patients and *H*. *pylori*

We utilized 64 isolates which was isolated from homogenized antral biopsy specimens of patients who underwent endoscopy examination at the Digestive Disease Center, Dr. Luis E. Aybar Health and Hygiene City, Santo Domingo, Dominican Republic in February 2011 [5]. Patients with a history of the antibiotic usage within 2 weeks prior to endoscopy, history of partial gastric resection, or history of previous treatment for *H*. *pylori* infection were excluded from the study. Importantly, all strains we used had information about resistance rate of standard *H*. *pylori* antibiotics (amoxicillin, clarithromycin, metronidazole, tetracycline and levofloxacin) from our previous study [5]. All *H*. *pylori* stock had been stored in Brucella Broth (Difco, NJ, USA) with 10% dimethyl sulfoxide and 10% horse serum at the -80°C. We also had information about *H*. *pylori* virulence factors (*cagA* and *vacA* genotypes), and about clinical outcomes which were diagnosed by endoscopic observation and histologic examination [5]. All participants submitted the written informed consent and we obtained study protocol approval from the ethics committees of Dr. Luis E. Aybar Health and Hygiene City, Institute of Microbiology and Parasitology, Autonomous University of Santo Domingo, Santo Domingo, the Dominican Republic; and the Oita University Faculty of Medicine, Japan.

### Antibiotic susceptibility testing

The MICs of five antibiotics including rifaximin (Tokyo Chemical Company, Tokyo, Japan), furazolidone (Tokyo Chemical Company, Tokyo, Japan), rifabutin (Sigma Aldrich, St. Louis, US), sitafloxacin (Haoyuan Chemexpress, Shanghai, China), and garenoxacin (Sigma Aldrich, US) were tested by using the two-fold agar dilution method. Prior the susceptibility test, the frozen stocks were subcultured twice on the Mueller-Hinton II agar contained 10% horse blood to recover the bacteria from the frozen environment and ensure the growth of the bacteria. The cultured bacteria were diluted in Brucella broth and were adjusted to the OD of 0.1. The prepared bacterial suspension was inoculated on Mueller-Hinton II agar contained with blood and antibiotic. Then the plates were incubated at 37°C under microaerophilic conditions (10% O2, 5% CO2, and 85% N2). The MICs were determined after 72-h incubation. As a quality control, an *H*. *pylori* strain from American Type Culture Collection (catalog #43504) was tested along with each batch of susceptibility test. The resistance breakpoint was determined on the MIC of each antibiotic tested: ≥4 μg/mL for rifaximin, ≥4 μg/mL for furazolidone, and ≥1 μg/mL for rifabutin, sitafloxacin, and garenoxacin, as previously described [14–17]. The rifaximin and furazolidone concentration were ranged between 0.25 μg/mL to 32 μg/mL, while the concentration range of rifabutin, sitafloxacin, and garenoxacin were between 0.064 μg/mL to 8 μg/mL.

### Molecular analysis of resistant strains

*H*. *pylori* genomic DNA was extracted by using commercial DNeasy Blood and Tissue kit (Qiagen, Hilden, Germany) and then stored at -20°C. The next-generation sequencing (MiSeq next-generation sequencer; Illumina, San Diego, CA) was used to analyze the full-length *rpoB*. The BLAST algorithm implemented in the CLC Genomic Workbench software (ver. 11; Qiagen, Venlo, Netherlands) was used to analyze the MiSeq output data. The sequences of *hp1198* of the strain 26695 were used as queries to obtain the *rpoB* sequence from the sequencing data. The variants in the *rpoB* were determined by comparing the full-length *rpoB* sequence of the strain 26695 (GenBank accession number AE000511.1) with the *rpoB* sequences of rifaximin-resistant strains and five randomly selected rifaximin-sensitive strain sequences. Then, all the sequences were aligned at the codon level by using MAFFT software, after confirming the absence of insertion or deletions leading to frameshift mutation. By comparing each codon of the resistant and sensitive strains, variants of codons were checked. The variants found in both sensitive and resistant strains were considered as normal variant. The variants found in the resistant strains but not in the sensitive strains were considered as variant-related to antibiotic resistance. In this study, we also utilized the data of the genetic mutations in the *gyrA* and *gyrB* from our previous study in Dominican Republic [5].

### Statistical analysis

We analyzed the discrete variables by the chi-square test, whereas the continuous variables by Mann-Whitney *U* and *t*-tests. A binary logistic regression model was used to calculate the odds ratio (OR). All determinants with P values of <0.10 were entered together in to the full logistic regression model, and the model was reduced by excluding variables with P values of >0.10. The OR and 95% confidence interval (CI) were used to estimate the risk. The statistically significant was determined by P values <0.05. All statistical analysis in this study was using SPSS statistical software package version 23.0 (SPSS, Inc., Chicago, IL).

## Results

### Five antibiotics susceptibility

**Fig 1:**
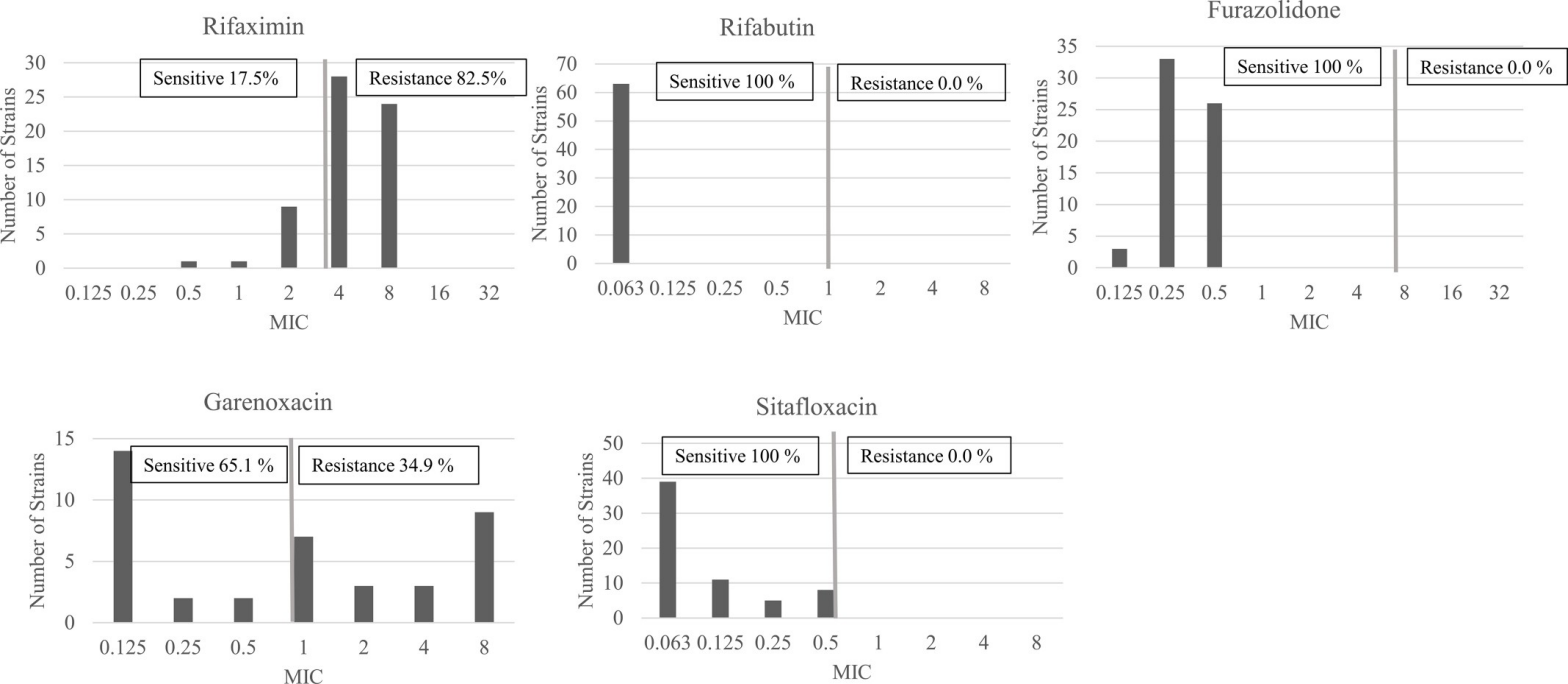
The distribution of alternative antibiotic susceptibility test result in Dominican Republic. The *H*. *pylori* resistance rate of rifaximin and garenoxacin were high in Dominican Republic. In contrast, all *H*. *pylori* isolate from Dominican Republic were sensitive towards rifabutin, furazolidone, and sitafloxacin.

Among 64 strains included, 63 *H*. *pylori* were successfully cultured (strain Dominica151 was not grown) and sub-cultured for agar dilution test including 19 strains from male and 44 from female patients. Rifaximin had extremely high prevalence of resistance (52/63, 82.5%). A high prevalence of garenoxacin resistance was also observed (22/63; 34.9%). In contrast, agar dilution test exhibited there was no resistant strain towards furazolidone, rifabutin, and sitafloxacin. The distribution of five antibiotics resistance pattern is shown in Table 1 and Fig 1. Male had higher prevalence of rifaximin resistance, but female had higher prevalence of garenoxacin resistance; however, there was no statistically significant association (both P >0.05). Patients more than or equal to 60 years old had the highest risk of garenoxacin resistances (8/9, 88.9%, OR = 45.33 [CI 4.055–506.836], P = 0.002). There was no association between antibiotic resistance and clinical outcomes (P >0.05). The correlation between age and the garenoxacin resistance based on the Spearman’s rank correlation model showed that an increasing age reflects the increased prevalence of garenoxacin resistance (P = 0.004, r = 0.357). The association between demographic and clinical outcome to rifaximin and garenoxacin of double-antibiotic resistance pattern is shown in Table 2. Eighteen strains had double-antibiotic resistance to rifaximin and garenoxacin (18/63; 28.6%). Patients more than or equal to 60 years old had the highest prevalence of double-antibiotic resistance (7/9, 77.8%, OR = 31.5 [CI 2.350–422.299], P = 0.009) than the patients less than 30 years old. There was no association between double-antibiotic resistance with sex and clinical outcome (P = 0.385 and P = 0.099, respectively). We also found a relatively high prevalence of quadruple-resistance strains (15/63; 23.8%). Almost all of the quadruple-resistance strains had resistance towards rifaximin, clarithromycin, levofloxacin, and metronidazole (14/15; 93.3%).

**Table 1:** Distribution of antibiotic resistance in the Dominican Republic.

Characteristic	N	Resistant Regimen (%)				
		Rifaximin	Furazolidone	Rifabutin	Garenoxacin	Sitafloxacin
Total	63	52/63 (82.5)	0/63 (0.0)	0/63 (0.0)	22/63 (34.9)	0/63 (0.0)
Sex						
Male	19	17/19 (89.5)	0/19 (0.0)	0/19 (0.0)	4/19 (21.0)	0/19 (0.0)
Female	44	35/44 (79.5)	0/44 (0.0)	0/44 (0.0)	18 (40.9)	0/44 (0.0)
Age						
<30	10	8/10 (80.0)	0/10 (0.0)	0/10 (0.0)	2/10 (20.0)	0/10 (0.0)
30–39	13	11/13 (84.6)	0/13 (0.0)	0/13 (0.0)	4/13 (30.8)	0/13 (0.0)
40–49	20	17/20 (85.0)	0/20 (0.0)	0/20 (0.0)	3/20 (15.0)	0/20 (0.0)
50–59	11	8/11 (72.7)	0/11 (0.0)	0/11 (0.0)	5/11 (45.4)	0/11 (0.0)
>59	9	8/9 (88.9)	0/11 (0.0)	0/11 (0.0)	8/9 (88.9)*	0/11 (0.0)
Clinical outcome						
Gastritis	47	41/47 (87.2)	0/47 (0.0)	0/47 (0.0)	19/47 (40.4)	0/47 (0.0)
PUD**	16	11/16 (68.8)	0/16 (0.0)	0/16 (0.0)	3/16 (18.8)	0/16 (0.0)

* P = 0.002, OR = 45.33 [CI 4.055–506,836]

** PUD; peptic ulcer disease

**Table 2:** Association between demographic and clinical outcome to rifaximin and garenoxacin double-antibiotic resistance in the Dominican Republic.

Characteristic	N	Rifaximin + Garenoxacin (%)	Crude OR	95% CI	P value
Total	63	18/63 (28.6%)			
Sex					
Male	19	4/19 (21.0%)	1		
Female	44	14/44 (31.8%)	1.750	0.490–6.246	0.389
Age					
<30	10	1/10 (10.0%)	1		
30–39	13	3/13 (23.1%)	2.700	0.236–30.846	0.424
40–49	20	3/20 (15.0%)	1.588	0.144–17.561	0.706
50–59	11	4/11 (36.4%)	5.143	0.465–56.897	0.182
>59	9	7/9 (77.8%)	31.500	2.350–422.299	0.009*
Clinical outcome					
Gastritis	47	16/47 (34.0)	3.613	0.730–17.890	0.116
PUD	16	2/16 (12.5)	1		

*P <0.05

**, PUD; peptic ulcer disease

### Susceptibility comparison with standard antibiotic

We compared our previous results with the resistance of five standard antibiotics (Table 3) [5]. Sitafloxacin, furazolidone, and rifabutin become a potential drug to be used as alternative *H*. *pylori* eradication antibiotic due to 100% sensitive especially as the second line in the high prevalence of levofloxacin and metronidazole resistance. Among 23 levofloxacin-resistant strains, almost all (19/23, 82.6%, P <0.001) were also resistant to garenoxacin, and 39.1% (9/23) had a high MIC ≥8 μg/mL with positive trend correlation (P = <0.001, r = 0.84). There was no association between the resistance of sitafloxacin and levofloxacin (P = 0.992). Although there was an association between the resistance of rifaximin and amoxicillin (P = 0.028), only one strain was resistant to amoxicillin. In contrast, there was no association between the resistance of rifaximin and clarithromycin, levofloxacin and metronidazole (P = 0.512, P = 0.991, and P = 0.945, respectively).

**Table 3:** Comparation susceptibility test of alternative vs. standard antibiotics.

Standard Regimen	Resistant rate (%)*	Alternative Regimen	Resistant rate (%)**
Clarithromycin	2/64 (3.1%)	Garenoxacin	22/63 (34.9%)
Amoxicillin	1/64 (1.6%)	Sitafloxacin	0/63 (0.0%)
Metronidazole	53/64 (82.8%)	Furazolidone	0/63 (0.0%)
Tetracycline	0/64 (0.0%)	Rifabutin	0/63 (0.0%)
Levofloxacin	23/64 (35.9%)	Rifaximin	52/63 (82.5%)

* This number is corresponded to our previous study

** This number is corresponded to our current study

### Multiple resistances in standard and alternative antibiotics

We combined the standard antibiotics susceptibility data from our previous study [5] with alternative antibiotics susceptibility. The pattern of multiple antibiotic resistance is shown in S1 Table. There was 1 strain that sensitive to all 10 antibiotics tested. Thirteen of 63 strains (20.6%) were resistant to only 1 antibiotic. Double antibiotic resistance was present in 25 strains (39.7%). Interestingly, there were 14 strains (22.2%) that had quadruple resistance towards rifaximin, garenoxacin, levofloxacin, and metronidazole. There was only 1 strain (1.6%) that had quintuple resistance towards rifaximin, garenoxacin, levofloxacin, and metronidazole, and clarithromycin.

### MIC and mutations among fluoroquinolone group

We compared the level of MIC between levofloxacin-resistant strains with the other fluoroquinolone group. Most of the strains possessing *gyrA* or *gyrB* mutations had considerably higher MIC level to levofloxacin (8–128 times higher) and garenoxacin (8–64 times higher), but around the same level with sitafloxacin. Among 19 garenoxacin resistant strains, 16 (84.2%) contained mutation at D91 and N87 (Table 4). Of nine garenoxacin resistant strains with high MIC ≥8 μg/mL, 8 (88.9%) contained an amino acid changes at 91 and 87 locations.

**Table 4:** Levofloxacin, garenoxacin, sitafloxacin and with *gyrA* and *gyrB* mutations.

Strains	*gyrA* mutation	*gyrB* mutation	Levofloxacin		Garenoxacin		Sitafloxacin	
			MIC	Status	MIC	Status	MIC	Status
Dominica08	None	None	0.25	S	<0.063	S	0.125	S
Dominica32	None	None	0.25	S	0.125	S	<0.063	S
Dominica57	None	None	0.25	S	0.125	S	<0.063	S
Dominica95	None	None	0.25	S	0.125	S	<0.063	S
Dominica9	None	None	16	R	8	R	0.5	S
Dominica10	N87I	None	32	R	8	R	0.5	S
Dominica14	D91G, E193D, I194F, A197F	None	2	R	1	R	0.125	S
Dominica18	N87T, D91N	None	16	R	8	R	0.5	S
Dominica43	N87T, D91N	None	2	R	0.5	S	0.125	S
Dominica44	N87T, A97V	None	16	R	8	R	0.25	S
Dominica49	N87A	D435N, S479G	32	R	8	R	0.5	S
Dominica50	E58G, G75V, D91G, Q98L	None	2	R	1	R	0.125	S
Dominica51	D91N	None	2	R	1	R	0.125	S
Dominica60	D91Y, A134V, D145N	None	2	R	0.5	S	<0.063	S
Dominica62	D91N	S479G	2	R	1	R	0.125	S
Dominica71	N87I	S479G	32	R	8	R	0.5	S
Dominica73	None	None	32	R	4	R	0.125	S
Dominica75	N87I	None	32	R	8	R	0.5	S
Dominica96	None	None	4	R	4	R	0.25	S
Dominica103	N87I, D91N, I162T	None	16	R	8	R	0.5	S
Dominica115	N87K	None	2	R	1	R	0.125	S
Dominica142	N87T, D91N	None	4	R	1	R	0.25	S
Dominica146	N87T	None	16	R	8	R	0.25	S
Dominica147	D91Y	None	2	R	2	R	0.125	S
Dominica148	None	None	2	R	0.25	S	<0.063	S
Dominica150	N87T, D91N, F149V	None	16	R	0.25	S	<0.063	S
Dominica152	N87I	Y514F	8	R	4	R	0.5	S

MIC: Minimal Inhibitory Concentration, in μg/mL. S: sensitive; R: resistant. N87I means isoleucine replaced asparagine in amino acid position 182. We ignored mutation that were present in both sensitive and resistant strains.

### Rifaximin resistance and *rpoB* mutations

We analyzed the *rpoB* sequence from 15 rifaximin-resistant strains (MIC ≥4 μg/mL). We found a total of 78-point mutations in *rpoB* categorized as missense mutation (S2 Table). All of the resistant strains possessed multiple mutations with more than 3 mutations within *rpoB*. We could not find mutation in codons 525 to 586 which was reported to have an association with rifamycin resistance in previous studies [12, 16, 18, 19]. Of the 15 strains, S352L and I2726V were the most common novel point mutations (6/15, 40.0% and 6/15, 40.0%, respectively, Table 5). V2465A point mutation appeared in four resistant strains (4/15, 26.7%), while A761V, M1628I, and T1929A point mutations appeared in 3 strains (3/15, 13.3%).

**Table 5:** The frequency of the 15 most common mutations in *rpoB*.

No.	Point Mutation	Total number (%)
1	S352L	6/15 (40.0)
2	I2726V	6/15 (40.0)
3	V2465A	4/15 (26.7)
4	A761V	3/15 (20.0)
5	M1628I	3/15 (20.0)
6	T1929A	3/15 (20.0)
7	S2889P	3/15 (20.0)
8	D2381E	2/15 (13.3)
9	A735T	2/15 (13.3)
10	D1163N	2/15 (13.3)
11	K1166R	2/15 (13.3)
12	S2525G	2/15 (13.3)
13	T2534M	2/15 (13.3)
14	A2884T	2/15 (13.3)
15	N2888G	2/15 (13.3)

S352L means leucine replaced serine in amino acid position 182. We ignored mutation that were present in both sensitive and resistant strains.

### Virulence genes and antibiotic resistance

We picked up the information of virulence factors (*cagA* and *vacA*) from our previous study [5]. The distribution and association between virulence genes and antibiotic is presented in Table 6. There was a significant association between *vacA* genotypes and rifaximin resistance (P = 0.033). The *vacA* s1/m1 was tended to have rifaximin resistance (P = 0.076) and garenoxacin resistance (P = 0.090). There was no significant association between *cagA* positivity with both rifaximin and garenoxacin resistance (P = 0.207 and P = 0.883, respectively).

**Table 6:** Association between virulence genes and resistance pattern.

Virulence genes	Rifaximin (%)			Garenoxacin (%)		
	S	R	P value	S	R	P value
Number of strains	11	52		41	22	
*cagA*			0.207			0.883
Positive	10/11 (90.9)	38/52 (73.1)		31/41 (75.6)	17/22 (77.3)	
Negative	1/11 (9.1)	14/52 (26.9)		10/41 (24.4)	5/22 (22.7)	
*vacA*						
s region			0.033*			0.116
s1*	11/11 (100)	36/52 (69.2)		28/41 (68.3)	19/22 (86.4)	
s2*	0/11 (0.0)	16/52 (30.8)		13/41 (31.7)	3/22 (13.6)	
m region			0.115			0.181
m1	10/11 (90.9)	35/52 (67.3)		27/41 (65.9)	18/22 (81.8)	
m2	1/11 (9.1)	17/52 (32.7)		14/41 (34.1)	4/22 (18.2)	
s1/m1	10/11 (90.9)	33/52 (63.5)	0.076	25/41 (61.0)	18/22 (81.8)	0.09
s1/m2	1/11 (9.1)	3/52 (5.8)	0.618	3/41 (7.3)	1/22 (4.5)	0.667
s2/m1	0/11 (0.0)	2/52 (3.8)	0.509	2/41 (4.9)	0/22 (0.0)	0.292
s2/m2	0/11 (0.0)	14/52 (26.9)	0.051	11/41 (26.8)	3/22 (13.6)	0.23

*P <0.05

### Nucleotide sequencing

Nucleotide sequence data from this study are available under DDBJ accession numbers LC425694–LC425711 (*rpoB*).

## Discussion

We confirmed the low prevalence of sitafloxacin resistance in the Dominican Republic. Moreover, all the strains possessed very low MIC, indicating that sitafloxacin was a potent antibiotic to be used as alternative antibiotic in the Dominican Republic. Our result was in agreement with previous studies reported that sitafloxacin were potential drugs to be included in *H*. *pylori* eradication regimen [20, 21]. Sitafloxacin was reported to be combined with other antibiotics, such as amoxicillin [20] and metronidazole [21] with relatively equal results [22]. However, due to the high prevalence of metronidazole-resistant strains in the Dominican Republic [5], it is not recommended to combine sitafloxacin with metronidazole. In contrast, the prevalence of amoxicillin-resistant strain is very low [5]. Thus, the combination of sitafloxacin and amoxicillin seems to be more reasonable to be used in the Dominican Republic. Currently, sitafloxacin is still not available in the Dominican Republic. But with its recent availability in Thailand after in Japan, hopefully it will be widely distributed. Sitafloxacin is already reported not only to be effective as *H*. *pylori* eradication therapy in Japan, but also for treatment of complicated urinary tract infection and pyelonephritis. Therefore, it might be important to provide sitafloxacin in the Dominican Republic.

Our study revealed all strains were sensitive towards rifabutin, supporting the possibility to recommend rifabutin as potential antibiotic to combat *H*. *pylori* infection. Previous study reported that rifabutin-based therapy was highly effective and reliable as an alternative therapy, especially after two times eradication failure [23, 24]. Rifabutin is known to be a broad spectrum of antimicrobial activity and has high activity towards varieties of Gram-positive and -negative bacteria, including Mycobacterium [11]. The effectiveness of rifabutin may be attributed to its basic physicochemical characteristics and increasing lipophilicity, tissue uptake, and intracellular concentration [25]. Rifabutin is reported to be absorbed better in the circulation and also a weak inducer of CYP450, resulting in better bioavailability in circulation [26]. A history of rifampicin usage, primarily in tuberculosis treatment should be taken into consideration before prescribing rifabutin for *H*. *pylori* eradication therapy. Combination of clarithromycin along with rifabutin should be considered carefully, as the combination between these antibiotics was showing the evidence of the inhibition of rifabutin metabolism, suggesting the possibility of toxicity [27]. Therefore, the combination of clarithromycin and rifabutin in the Dominican Republic should be carefully prescribed, even though the clarithromycin-resistant strain was low [5].

Similar to sitafloxacin and rifabutin, furazolidone resistance showed to be low in the Dominican Republic. Furazolidone is one of the antibiotic recommended by several guidelines to be included in eradication regimen [8, 28]. Furazolidone is included in quadruple-therapy regimen, substituting metronidazole and combined with the addition of bismuth [29] to increase the efficacy of the therapy. Although the bismuth-quadruple containing furazolidone was shown to be effective [30, 31], the unavailability of bismuth make this regimen could not be applied in many population.

Levofloxacin resistance is widely known to be associated with the mutations in *gyrA* and/or *gyrB*. Our previous study also mentioned that more than third-quarter of the levofloxacin-resistant strains in the Dominican Republic had amino acid substitution associated with *gyrA* mutations [5]. Our study showed that despite the occurrence of mutations in *gyrA*, sitafloxacin was still highly effective. This result indicates that mutations in these genes had less effect on sitafloxacin susceptibility than levofloxacin. Our result also in concordance with previous study in Japan which reported the superior antibacterial activity of sitafloxacin against *H*. *pylori* compared to that of garenoxacin and levofloxacin even in the presence of mutations in *gyrA* [15]. This result was also in concordance with previous study which reported that sitafloxacin might overcome the antibiotic resistance of *H*. *pylori* with *gyrA* mutation [9]. Although garenoxacin is included in the same fluoroquinolone with sitafloxacin without significant side effect, we found the high prevalence of garenoxacin resistance. Suggesting a less effect of garenoxacin to inhibit the gyrase function, thus, it may not suitable as second line regimen in the Dominican Republic. The increasing drug resistance in the older age might be related to the improper antibiotic usage and also the spreading of resistant strain in their environment such as nursing home, which might be a reservoir for multidrug-resistance strain [32].

Compared to other four antibiotics tested in this study, rifaximin was shown to be the one with exceptionally very high resistance prevalence. Although our study revealed low susceptibility rate of rifaximin as single-antibiotic therapy, a better result was obtained by combining rifaximin with another antibiotic, such as clarithromycin [10, 33, 34]. The remarkable safety of rifaximin allows the high-dose regimen or longer duration of therapy, make it a promising drug to be used as alternative antibiotic in *H*. *pylori* eradication regimen and to be tested in clinical trial phase [10]. Moreover, rifaximin might become a potential drug to be used to combat *H*. *pylori* infection in childhood, due to the high safety properties [35, 36]. While rifaximin resistance was high in the Dominican Republic, further study is needed to examine the efficacy of rifaximin-base combination therapy in clinical trial.

In this study, we analyzed the mutation of the genes related to the antibiotic resistance. The *rpoB* mutations is widely known to have important role in the rifamycin-group drug resistance mechanism in various microorganism, such as *Clostridium difficile* [37], *Mycobacterium tuberculosis* [38–40], and *Escherichia coli* [41]. In this study, we found the occurrence of more than 3 mutations within *rpoB* which was rarely reported. In addition, although numerous mutations in *rpoB*, most of them only appeared in one resistant strain including novel mutations; S352L, I2726L, and V2465A, suggesting molecular antibiotic susceptibility testing is not applicable for rifaximin. Interestingly, in this study we found that there was no resistant strain towards rifabutin even though the resistance towards rifaximin was high, while previous studies mentioned that the *rpoB* mutations was associated with rifamycin resistance [12, 19]. However, our result is in concordance with previous study in Japan which mentioned that *rpoB* mutation-positive strain showed successful *H*. *pylori* eradication by using rifabutin-based therapy [42]. This phenomenon also occurs in other bacteria, as previous study reported that Mycobacterial strains bearing the mutation is *rpoB* is often rifabutin-susceptible [43]. Our previous study in Dominican Republic reported the association between antibiotic resistance and the genes mutations involved in the antibiotic resistance (e.g. *rdxA* and metronidazole resistance, 23S rRNA and clarithromycin resistance) (PMIID: 28193745). As expected, when we analyzed the association between *rdxA*, 23S rRNA and PBP-1A with the *H*. *pylori* resistance to garenoxacin and rifaximin, we confirmed that there was no association between the mutations in PBP-1A, *rdxA*, and 23S rRNA to garenoxacin and rifaximin resistance (data not showed). As for our proposed treatment regimen in this current study, we observed no *H*. *pylori* resistant to sitafloxacin, rifabutin and furazolidone; therefore, even we observed mutations related to metronidazole in *rdxA* gene, levofloxacin resistance in *gyrA* and *gyrB* genes as well as clarithromycin resistance in 23S rRNA gene, those mutations were not affecting the sitafloxacin, rifabutin and furazolidone resistance.

There are several limitations in this study. First, sample number is relatively small, and we collected samples only from the capital city of the Dominican Republic, thus our results could not be generalized across Latin America. However, we believe that our current data would become important data to decide a general policy for eradicating *H*. *pylori* in this region. Second, out of thousands *H*. *pylori* genes, we only analyzed few genes that might be related to antibiotic resistance mechanism. Since we have large data from our next-generation sequencing, detailed genome-wide association study of *H*. *pylori* genome is now in progress. Third, we only determined resistance rate of five alternative antibiotics by in vitro study. Clinical trial should be required to examine the efficacy of five alternative antibiotics-based combination therapy.

## Conclusions

Sitafloxacin, rifabutin, and furazolidone might be considered as an alternative antibiotic to be included in the therapy to *H*. *pylori* eradication regimen in regions with high prevalence of levofloxacin and metronidazole resistance such as the Dominican Republic.

## Supporting information

S1 Table Antibiotic resistance combinations of *H. pylori* isolate from Dominican Republic. (DOCX)

S2 Table Mutations in *rpoB* that was associated with rifaximine resistance. (DOCX)
