# Supplementary material for: Five alternative Helicobacter pylori antibiotics to counter high levofloxacin and metronidazole resistance in the Dominican Republic
Source: PLoS One. 2019 Mar 27;14(3):e0213868. doi: 10.1371/journal.pone.0213868 (PMC6436749; doi:10.1371/journal.pone.0213868)
Supplement: S1 Table — (DOCX) [file pone.0213868.s001.docx]

**S1 Table. Antibiotic resistance combinations of *H. pylori* isolate from Dominican Republic**

| **Susceptibility test result** | **Number**  **(Total = 63)** | **Prevalence (%)** |
| --- | --- | --- |
| **Sensitive for all*** | 1 | 1.6 |
|  |  |  |
| **Single resistance only** | 13 | 20.6 |
| RFX | 7 | 11.1 |
| FUR | 0 | 0 |
| RIB | 0 | 0 |
| GAR | 1 | 1.6 |
| SIT | 0 | 0 |
| CAM** | 0 | 0 |
| AMX** | 0 | 0 |
| MNZ** | 5 | 7.9 |
| TCN** | 0 | 0 |
| LVX** | 0 | 0 |
|  |  |  |
| **Double resistance only** | 25 | 39.7 |
| RFX+GAR | 1 | 1.6 |
| RFX+CAM | 0 | 0 |
| RFX+AMX | 0 | 0 |
| RFX+MNZ | 23 | 36.5 |
| RFX+LVX | 0 | 0 |
| GAR+CAM | 0 | 0 |
| GAR+AMX | 0 | 0 |
| GAR+MNZ | 0 | 0 |
| GAR+LVX | 0 | 0 |
| CAM+AMX | 0 | 0 |
| CAM+MNZ | 0 | 0 |
| CAM+LVX | 0 | 0 |
| AMX+MNZ | 0 | 0 |
| AMX+LVX | 0 | 0 |
| MNZ+LVX | 1 | 1.6 |
|  |  |  |
| **Triple resistance only** | 8 | 12.7 |
| RFX+GAR+MNZ | 1 | 1.6 |
| RFX+GAR+LVX | 1 | 1.6 |
| RFX+CAM+MNZ | 1 | 1.6 |
| RFX+LVX+MNZ | 3 | 4.8 |
| GAR+LEV+MNZ | 2 | 3.2 |
|  |  |  |
| **Quadruple resistance only** | 15 | 23.8 |
| RFX+GAR+LEV+MNZ | 14 | 22.2 |
| GAR+AMX+LEV+MNZ | 1 | 1.6 |
|  |  |  |
| **Quintuple resistance only** | 1 | 1.6 |
| RFX+GAR+CAM+LEV+MNZ | 1 | 1.6 |
| Abbreviations: AMX, amoxicillin; CAM, clarithromycin; MNZ, metronidazole; TCN, tetracycline; LVX, levofloxacin; GAR, garenoxacin; SIX, sitafloxacin; FUR, furazolidone; RIB, rifabutin; RFX, rifaximine | | |
| * 1 strain that is sensitive to RFX, FUR, RIB, GAR, SIT, CAM, AMX, MNZ, TCN, and LVX. | | |
| ** This number is corresponded to our previous reports of the same strains, which is referring to CAM, AMX, MNZ, TCN, and LVX resistances | | |
