# Supplementary material for: Five alternative Helicobacter pylori antibiotics to counter high levofloxacin and metronidazole resistance in the Dominican Republic
Source: PLoS One. 2019 Mar 27;14(3):e0213868. doi: 10.1371/journal.pone.0213868 (PMC6436749; doi:10.1371/journal.pone.0213868)
Supplement: S2 Table — (DOCX) [file pone.0213868.s002.docx]

**S2 Table. Mutation in *rpoB* that was associated with rifaximin resistance**

| **No** | **Strain** | **MIC (µg/mL)** | **Mutation list** |
| --- | --- | --- | --- |
| 1 | Dominica08 | 8 | R182K, S352L, D1163N, K1166R, M1628I, T1929A |
| 2 | Dominica10 | 8 | V779I, R1173H, A1382V, G2337S |
| 3 | Dominica18 | 8 | M494V, R2365C, P2546S, G2601E, V2639I, I2726V |
| 4 | Dominica30 | 8 | S352L, M1628I, A1890V, A1948V, V2465A, S2489G, A2511V, S2525G, T2534M, I2726V, A2884T, N2888G, S2889P |
| 5 | Dominica32 | 8 | E870G, D968N, G1979S, D2381E, K2422R, V2465A, D2500G, R2642K |
| 6 | Dominica49 | 4 | M415V, A761V, T1929A, A2358V |
| 7 | Dominica51 | 8 | S352L, A761V, A2101V, K2344E, T2537A, F2538L, K2539S, I2726V, V2803M, A2884T, S2887Y, N2888D, S2889P, F2891I |
| 8 | Dominica52 | 4 | A198T, T886A, A959T, L1508F, A1644V, N1761D, N2603H, I2726V, A2884V |
| 9 | Dominica57 | 8 | A735T, L1062F, V2465A, K2692R, I2726V |
| 10 | Dominica60 | 8 | Y70C, M86I, S352L, P636L, A735T, R812H, V986I, S2391G |
| 11 | Dominica64 | 8 | T431N, A732T, A954V |
| 12 | Dominica84 | 8 | R812C |
| 13 | Dominica95 | 8 | D61N, A872V, K1230N, I2589V, S2623V, I2726V |
| 14 | Dominica116 | 4 | S352L, D1163N, K1166R, I1548V, M1628I, R1683H, T1929A, R2776H |
| 15 | Dominica157 | 4 | A244T, K307E, S352L, A761V, S1277A, D2381E, V2465A, S2525G, T2534M, H2710Y, N2888G, S2889P |

MIC = minimum inhibitory concentration. R182K means lysine replaced arginine in amino acid position 182. We ignored mutation that were present in both sensitive and resistant strains.
